# Insights on Structural, Mechanical and Thermal Properties of High-Entropy Perovskite Oxide (Ca_0.2_Sr_0.2_Ba_0.2_La_0.2_Pb_0.2_)TiO_3_ from First-Principles Calculations

**DOI:** 10.3390/ma19091845

**Published:** 2026-04-30

**Authors:** Lin Shao, Shuaiqi Liu, Pingying Tang, Riwen Ji

**Affiliations:** 1 Guangxi Key Laboratory of Natural Polymer Chemistry and Physics, College of Chemistry and Material Science, Nanning Normal University, Nanning 530001, China; shaolin@nnnu.edu.cn (L.S.); 19854680780@163.com (S.L.); 2 Guangxi Key Laboratory of Functional Information Materials and Intelligent Information Processing, Nanning Normal University, Nanning 530001, China; tangpy@nnnu.edu.cn; 3 Guangxi Zhuang Autonomous Region Institute of Product Quality Inspection, Nanning 530007, China

**Keywords:** high-entropy perovskite, local lattice distortion, elastic parameters, thermal conductivity, electronic structure

## Abstract

High-entropy perovskite oxides attract considerable attention due to their outstanding properties and extensive applications. In this work, the lattice distortion and the mechanical, thermal and electronic structure properties of high-entropy (Ca_0.2_Sr_0.2_Ba_0.2_La_0.2_Pb_0.2_)TiO_3_ (CSBLPT) are investigated through first-principles calculations. The results suggest that the influence of O atoms on lattice distortion is predominant, and the effect of overall A-site atoms plays a distinctly greater role than that of the B-site atoms. The mechanical results show that the high-entropy CSBLPT has a lower Young’s modulus and higher fracture toughness than ternary SrTiO_3_. The Debye temperature also indirectly indicates that the thermal expansion coefficient of the studied high-entropy perovskite is greater than that of SrTiO_3_. As for thermal conductivity, the obtained result of CSBLPT is also appreciably lower than that of SrTiO_3_, and the lowest thermal conductivity is along the [100] direction. The Fermi level of high-entropy CSBLPT is transferred to the conduction band, exhibiting a degenerate n-type semiconductor behavior with metallic-like characteristics, and the Bader charge values are also related to the local lattice distortion, which may cause differences in thermomechanical properties between high-entropy CSBLPT and SrTiO_3_. Above all, high-entropy CSBLPT is a preferable TBC material with excellent performance under working conditions compared to SrTiO_3_.

## 1. Introduction

Thermal barrier coatings (TBCs) can effectively protect thermal components of aircraft engines and assist the metal substrate to serve safely in harsh, high-temperature environments because of their excellent thermal insulation and high-temperature oxidation resistance [1,2]. Thus, TBC technology is regarded as one of the three core technologies for advanced aircraft engine turbine blades. For decades, 6–8 wt% Y_2_O_3_-stabilized ZrO_2_ (YSZ) has been considered the industry standard for TBC materials. However, due to its phase instability and high sintering rate, the service capacity of YSZ was limited to below 1200 °C [3,4]. Therefore, it is crucial to explore new oxide compositions with lower thermal conductivity and superior thermal stability as an alternative to conventional TBCs.

Among the extensively studied refractory metallic oxides [5,6,7,8,9], perovskite oxides (ABO_3_) with complex crystal structures are also prospective TBC ceramic materials due to their low thermal conductivity at high temperatures and good chemical stability. For example, the thermal conductivity of SrZrO_3_ and CaZrO_3_ at 1000 °C is 2.08 W∙m^−1^∙K^−1^ and 2.1 W∙m^−1^∙K^−1^, respectively [10,11], lower than that of YSZ. Nevertheless, there are also some ternary ABO_3_-type perovskites, like SrTiO_3_, that have low minimum thermal conductivities κ_min_ (1.74 W∙m^−1^∙K^−1^) but relatively high thermal conductivities (12 W∙m^−1^∙K^−1^) near room temperature [12,13], which hinders their practical application.

Since the concept of multi-principal high-entropy alloys was introduced, it has been found that their unique multi-component ratios and chemically disordered structures give the material unprecedented high entropy effects, and the subsequently reported rock-salt structured (Mg_0.2_Co_0.2_Ni_0.2_Cu_0.2_Zn_0.2_)O oxide [14] also provided new insights to open up research on high-entropy non-metallic materials. Therefore, based on studies of SrTiO_3_-based ceramics, high entropy can be introduced into its structure to reduce its thermal conductivity by effectively scattering phonons. Zheng et al. [15] have developed high-entropy perovskite ceramic (Ca_0.2_Sr_0.2_Ba_0.2_La_0.2_Pb_0.2_)TiO_3_ (abbreviated as CSBLPT) with A-site equal molar ratio substitution by solid-state reaction and conventional sintering, and reported the significant suppression of thermal conductivity of SrTiO_3_-based ceramics by using a high entropy strategy. Zhang et al. [16] have explored the detailed structural properties of (Ca_0.2_Sr_0.2_Ba_0.2_La_0.2_Pb_0.2_)TiO_3_ high-entropy perovskite, and also further verified that it exhibits a lower thermal conductivity and higher Seebeck coefficient. Although some progress has been made on high-entropy CSBLPT with perovskite structures, the current research is mainly focused on experimental methods for their synthesis and performance evaluation. Moreover, their excellent comprehensive properties [17,18,19] are mainly derived from their own flexible compositions and special structures. Then, how to evaluate the properties of high-entropy perovskite at the atomic level and comprehensively grasp the structure–property relationship is of great significance for further understanding of high-entropy perovskites.

For the TBCs, the intrinsic mechanical/thermal properties and their mechanisms are important bases for evaluating whether their high reliability and durability are suitable for TBC applications. Based on the perovskite-type SrTiO_3_, the present work proposes to carry out a study on the performance of high-entropy perovskite CSBLPT as TBC material. The structural characteristics of the substance were studied in detail at the atomic level by using first-principles calculations based on the density functional theory (DFT) method, and its influence on the structural stability and mechanical and thermal properties is further revealed. Finally, the electronic structures are studied to explore the influence of the high entropy effect on their thermal properties. This research provides important theoretical support for the practical application of high-entropy CSBLPT in TBCs.

## 2. Calculation Methods and Details

The experimental XRD result from Ref. [16] reveals that high-entropy CSBLPT perovskite inherited the cubic perovskite phase (Pm-3m) of traditional SrTiO_3_, and the multi-metal elements randomly entered the A-site lattice of SrTiO_3_ to form a single-phase disordered solid solution. To better mimic the complete chemical disorder in the sublattice, the special quasi-random structure (SQS) method [20], as implemented in the Alloy Theoretic Automated Toolkit (ATAT 3_50) code [21], was adopted to construct the chemical disorder of the multi-component CSBLPT structure, and the SQS supercells were expanded to 3 × 3 × 5, containing 225 atoms. All calculations were carried out in first-principles calculations by utilizing the Projector-Augmented Wave (PAW) methods within the framework of density functional theory (DFT) [22,23] and using the Vienna ab initio Simulation Package (VASP) version 5.4.4. The exchange–correlation interactions were dealt with using the generalized gradient approximation (GGA) of revised Perdew–Burke–Ernzerhof (PBEsol) [24]. For high-entropy CSBLPT perovskite, the cut-off energy of the plane wave was set as 520 eV, and the Brillouin zone integration of 4 × 4 × 2 k-points mesh based on the Monkhorst–Pack scheme was used. The convergence criterion of electronic energy and ionic Hellmann–Feynman force were less than 10^−4^ eV and 10^−2^ eV/Å, respectively, in all the DFT optimizations, and all components of the stress tensor, except those conjugate to the imposed strain, were converged to within 0.05 GPa. In the present work, all DFT calculations were performed on high-performance computing clusters equipped with Intel Xeon Platinum 8488C processors and 256 GB RAM per node, and the calculations were run in parallel using 40 cores for each job.

## 3. Results and Discussion

### 3.1. Lattice Structure

From the experimental results [16], the high-entropy perovskite CSBLPT can be regarded as a solid solution formed by SrTiO_3_, CaTiO_3_, BaTiO_3_, LaTiO_3_ and PbTiO_3_, and it exhibited a single cubic phase with space group Pm-3m. To investigate ground-state properties, the structural stability of high-entropy and corresponding ternary perovskites is first predicted by the tolerance factor t [25]. From Table 1, all the calculated t values of perovskites are approximately equal to 1, indicating that cubic-phase perovskites will be formed. Similar to high-entropy alloys, we evaluated the A-site cation size disorder of the CSBLPT perovskite with atomic size differences δ [26] and present the calculated results in Table 1. Although the cation size difference does not seem to be an important factor in determining the formation of a single high-entropy perovskite phase, as shown by Jiang et al. [26], a smaller δ can still demonstrate that it is easier to form a single-phase solid solution. The obtained δ value of 6.73% for CSBLPT perovskite is far lower than 13.3% reported by Jiang et al. [26], suggesting that the high-entropy perovskite can be formed in terms of size disorder.

Subsequently, the equilibrium lattice parameters of fully relaxed structures for CSBLPT and corresponding ternary perovskites are obtained by calculating and fitting the total energy *E* as a function of volume *V* according to the Birch–Murnaghan equation of states, and the calculated results and other available experimental and theoretical data are listed in Table 1. As can be seen, the obtained lattice parameters of CSBLPT perovskites match well with the relevant experimental data. Compared with the ternary perovskites, it can be seen that the lattice parameter of high-entropy perovskite is greater than that of SrTiO_3_, CaTiO_3_ and LaTiO_3_, but smaller than that of BaTiO_3_ and PbTiO_3_. Moreover, the tolerance factor *t* of high-entropy perovskite corresponds to the weighted average *t* of the corresponding ternary material, which suggests that the elements of Sr, Ca, Ba, La and Pb are uniformly occupied in the A-site and form a single-phase solid solution.

**Figure 1:**
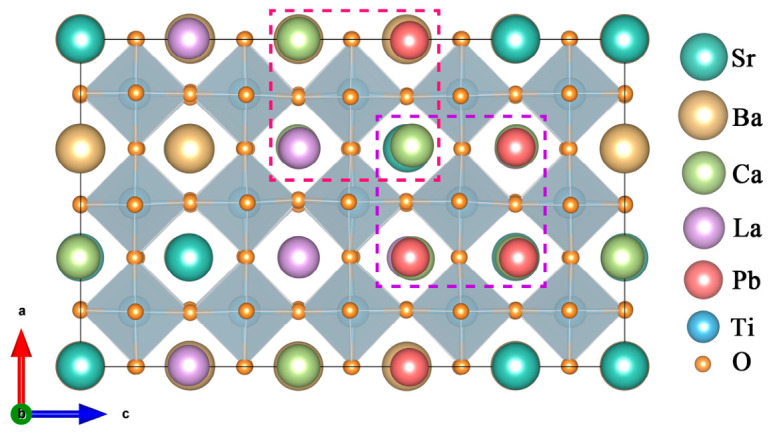
The supercell structure of high-entropy perovskite (Ca_0.2_Sr_0.2_Ba_0.2_La_0.2_Pb_0.2_)TiO_3_ with distortion.

In high-entropy CSBLPT, the significant differences in ionic radii and valence exist among Ca, Sr, Ba, La and Pb at the A-site [32], which will inevitably lead to the non-uniformity of local stress and charge distribution. Therefore, the high-entropy CSBLPT structure shows more obvious local lattice distortion, as seen in Figure 1. Moreover, as one of the four core characteristics of high-entropy ceramics, the lattice distortion can be adjusted by modulating the chemical disorder in the structure. In order to assess the magnitudes of lattice distortion for constituent atoms inside the structures, it was first quantitatively and rationally described by atomic displacements Δ*d* [33], which can be derived from the magnitude of deviation between the relaxed atomic position and its ideal position in the cubic lattice, and the results are listed in Table 2. It can be noticed that the overall average atomic displacement of A-site, Ti and O atoms are 0.056, 0.031 and 0.081, respectively, indicating that the influence of O atoms on lattice distortion is predominant, and the effect of overall A-site atoms plays a distinctly greater role than that of the B-site atoms.

**Figure 2:**
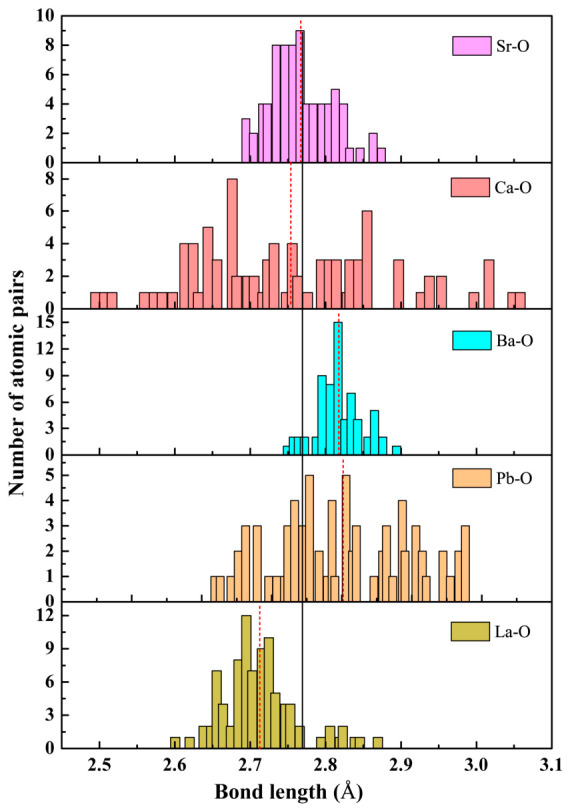
The bond length distribution of various A–O bonds for high-entropy CSBLPT. The ideal positions are indicated with black solid lines, and the average bond length with distortion is indicated with red dashed lines.

In addition, the chemical bonding in the structure is closely associated with most physicochemical properties of the material, including mechanical strength, thermal conductivity and electronic behavior. Thus, it can also quantitatively assess the magnitude of structural distortion in terms of the bond length distribution. For high-entropy CSBLPT, the A-site with multi-metals is randomly filled in the central position of TiO_6_ octahedra and naturally results in a large degree of disorder, so the bond length distributions of various A–O bonds in the distorted structure are obtained and shown in Figure 2. The black solid vertical line indicates the ideal A–O bond length calculated from the equilibrium lattice parameter of the undistorted cubic lattice (2.769 Å for CSBLPT). The red dashed lines indicate the average bond lengths after structural relaxation. It is apparent that the average bond length between constituent metals at A-sites and O atoms deviated from the ideal position and exhibited a wide range of distribution. Furthermore, combining the results in Table 2 also shows that the changes in bond length for Ca–O and Pb–O bonds originate mainly from the displacement of the metal ions, while the changes in the other three types of A–O bonds are mainly related to the contribution from the displacement of O atoms.

### 3.2. Mechanical Property

The mechanical properties are the macroscopic behavioral characteristics manifested in the process of deformation or fracture damage of the material under the application of external forces, and represent an important basis for evaluating whether the TBC materials have reliable durability, which can be described in terms of elastic and fracture properties. In order to check the mechanical stability of high-entropy perovskite in the ground state, the energy–strain method is implemented to obtain three independent single-crystal elastic constants (*C*_11_, *C*_12_ and *C*_44_) of the studied perovskites with cubic structure. The polycrystalline elastic moduli and other relevant parameters, such as bulk modulus *B*, shear modulus *G*, Young’s modulus *E* and Poisson’s ratio, *v* can be determined according to the Voigt–Reuss–Hill approximation [34].

The single-crystal elastic constants, polycrystalline elastic moduli and other relevant mechanical parameters are summarized in Table 3. From Table 3, one can see that the high-entropy CSBLPT and ternary matrix perovskite SrTiO_3_ are mechanically stable against elastic deformation because of the compliance of the obtained single-crystal elastic constants with the Born mechanical stability criterion for cubic symmetry *C*_11_ > |*C*_12_| and *C*_11_ + 2*C*_12_ > 0, *C*_44_ > 0. Also, the calculated elastic parameters of SrTiO_3_ are in reasonable agreement with experimental and other theoretical values [12,35]. By comparing the matrix SrTiO_3_, the CSBLPT perovskite still has high resistance to volume deformation and shear deformation, despite the reduction in elastic constants and modulus for the formed high-entropy perovskite. It is worth noting that the high-entropy CSBLPT exhibits a lower Young’s modulus compared to SrTiO_3_, suggesting that this high-entropy substance is more suitable as a TBC material than ternary SrTiO_3_, which is due to the fact that TBC materials with a lower Young’s modulus can improve the strain tolerance in service, thereby achieving better thermo-mechanical stability and improving the thermal cycling life of TBC [5,36].

Poisson’s ratio *v* and Pugh’s ratio *G*/*B* are significant elastic parameters to evaluate the ductile/brittle behavior of mechanical performance for materials, and the magnitude of the value indicates the degree of brittleness or ductility. From Table 3, the calculated Poisson’s ratio *v* of SrTiO_3_ is consistent with the other calculation results [12] and less than the boundary value of 0.26 for determining ductile behavior, indicating that it is a brittle material. However, the *v* of the high-entropy CSBLPT is significantly greater than that of the ternary matrix SrTiO_3_ and higher than 0.26, which reveals that the introduced high entropy effect improves the ductile behavior of CSBLPT perovskite and enhances its plasticity. Similarly, the threshold value of Pugh’s ratio *G*/*B* is about 0.57 to distinguish the brittleness or ductility of materials. The obtained *G*/*B* value of less than 0.57 also demonstrates that CSBLPT perovskite has ductile characteristics. These results may be due to lattice distortion effects in high-entropy materials; thus, a proper understanding of the lattice distortion in high-entropy materials is important to reveal the unique toughening behavior in high-entropy alloys.

**Figure 3:**
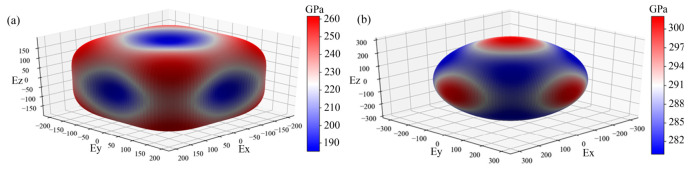
The 3D surface profiles of the orientation-dependent Young’s modulus of high-entropy CSBLPT (**a**) and ternary matrix SrTiO_3_ (**b**).

The Zener anisotropy index *A*_Z_ can be used to characterize the degree of elastic anisotropy in the lattice structure. From the obtained *A*_Z_ in Table 3, high-entropy CSBLPT and ternary SrTiO_3_ are anisotropic, and CSBLPT perovskite has greater elastic anisotropy due to the larger deviation of the Zener index from 1. Moreover, the derived data listed in Table 4 apparently show that *G*_[100]_ ≠ *G*_[110]_ ≠ *G*_[111]_ and *E*_[100]_ ≠ *E*_[110]_ ≠ *E*_[111]_, also proving the existence of elastic anisotropy in both investigated materials. Additionally, the *A*_Z_ of the cubic structure also determined the direction of the maximum Young’s modulus (*E*_max_) displayed in the lattice. For Az < 1, *E*_max_ will appear along the [100] direction, and conversely, *E*_max_ will occur in the [111] direction. Hence, it can be deduced that the *E*_max_ of high-entropy CSBLPT appears along the [111] direction, while that of ternary SrTiO_3_ is along the [100] direction, and the results are also confirmed in Table 4. Simultaneously, the 3D surface profiles of the orientation-dependent Young’s modulus of both substances are also displayed in Figure 3, from which this conclusion can again be supported.

The fracture toughness also contributes to the assessment of the mechanical properties of TBC materials, which is expressed by the formula $K_{IC}=V_{0}^{\frac{1}{6}}G{\left(B/G\right)}^{\frac{1}{2}}$ [37]. In general, ceramic materials with high fracture toughness and low Young’s modulus are conducive to preventing crack propagation and improving the thermal cycle life of TBCs. The calculated *K*_IC_ value of high-entropy CSBLPT is 4.16 MPa·m^1/2^, clearly higher than that of SrTiO_3_, which may be due to the incorporation of multiple cations in the A-site, improving the overall mixing entropy of the material and increasing the lattice distortion of the structure, thus enhancing the resistance to crack extension. Therefore, the result further suggests that high-entropy CSBLPT can be used as a potential material for TBCs.

### 3.3. Thermal Properties

Thermal properties are the most important performance index of TBC materials, being of great significance to the optimal design, production and practical application of material composition, and are also one of the basic parameters of theoretical research. Thus, the thermal properties of high-entropy CSBLPT as a TBC material are further explored by combining first-principles with the quasi-harmonic Debye–Grüneisen model.

The Debye temperature, as an important indicator of the thermal properties of a material, is related to both the heat capacity and the coefficient of thermal expansion, which can be estimated from the elastic constant according to the formula in Ref. [38]. As can be shown in Table 5, the high-entropy CSBLPT has a lower Debye temperature than that of ternary matrix SrTiO_3_, indicating a weaker interatomic interaction in high-entropy CSBLPT, from which it can be inferred that the thermal expansion coefficient of the studied high-entropy perovskite is greater than that of ternary matrix SrTiO_3_, mainly because of weaker interatomic forces, and thus the atoms are more likely to vibrate and displace when subjected to heat, resulting in a relatively greater thermal expansion coefficient of the material. The ceramic material with a greater thermal expansion coefficient has a longer service life due to a better match with the metal substrate at high temperatures. Therefore, CSBLPT is more suitable as a TBC material than SrTiO_3_.

Thermal conductivity is a measure of a material’s ability to conduct heat and can be essential for finding potential TBC materials. The theoretical minimum high-temperature thermal conductivity *κ*_min_ is the lowest limit of thermal conductivity value defined for temperatures much higher than the Debye temperature, which can be respectively predicted using Clarke’s model [39] and Cahill’s model [40] with the following equations:

(1) $$\kappa_{\min}^{Clarke}=0.87k_{B}{\left(\frac{M}{n\rho N_{A}}\right)}^{-\frac{2}{3}}\sqrt{\left(\frac{E}{\rho }\right)}$$

(2) $$\kappa_{\min}^{Cahill}=\frac{k_{B}}{2.48}{\left(\frac{n}{V}\right)}^{\frac{2}{3}}\left(v_{l}+2v_{t}\right)$$

The predicted minimum thermal conductivity of CSBLPT and SrTiO_3_ is shown in Table 5. It can be seen that the thermal conductivity of CSBLPT calculated with Clarke’s model and Cahill’s model is about 18.5% and 18.7% smaller than that of SrTiO_3_, respectively, which is mainly due to the multicomponent elements and heavier average mass of the A-site atoms in the high-entropy CSBLPT, and the local stress field caused by greater lattice distortion promotes the multi-scale scattering of phonons, resulting in increased phonon scattering. Phonon scattering is one of the most important factors affecting the thermal conductivity of a material; thus, the greater phonon scattering reduces the thermal conductivity of high-entropy CSBLPT compared to ternary SrTiO_3_. Furthermore, our calculations follow the rule that materials with lower Debye temperatures also have lower minimum thermal conductivity. This combination is highly desirable for TBC applications, where both good thermal expansion matching and low thermal conductivity are required. Therefore, the high-entropy CSBLPT perovskites evaluated in this work are thought to be a promising candidate for TBC material.

**Figure 4:**
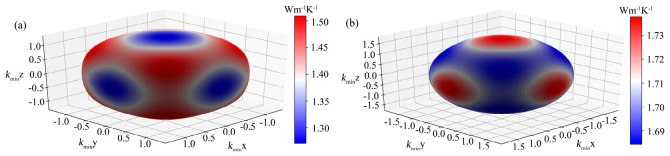
The 3D surface profiles of the orientation-dependent minimum thermal conductivity of high-entropy CSBLPT (**a**) and ternary matrix SrTiO_3_ (**b**).

As a TBC material, the anisotropy of minimum thermal conductivity is also worth further exploring. According to both models, bonding anisotropy will lead to anisotropy of elastic constants, and ultimately, this can be reflected as anisotropy in minimum thermal conductivity. Therefore, the 3D surface profiles of the orientation-dependent minimum thermal conductivity for both perovskites can be calculated, as shown in Figure 4 (take Clarke’s model as an example). One can see that the lowest minimum thermal conductivity is along the [100] direction for CSBLPT and along the [111] direction for SrTiO_3_, while the highest one is along the [111] direction for CSBLPT and along the [100] direction for SrTiO_3_. This variation is similar to the surface profile characteristic of Young’s modulus anisotropy.

### 3.4. Electronic Structure

**Figure 5:**
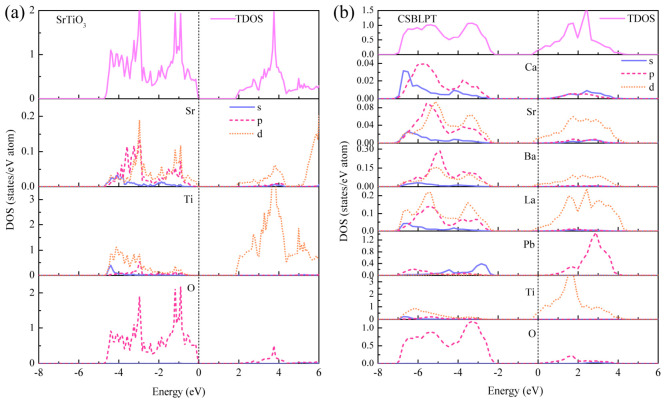
The density of state for ternary matrix SrTiO_3_ (**a**) and high-entropy CSBLPT (**b**).

**Figure 6:**
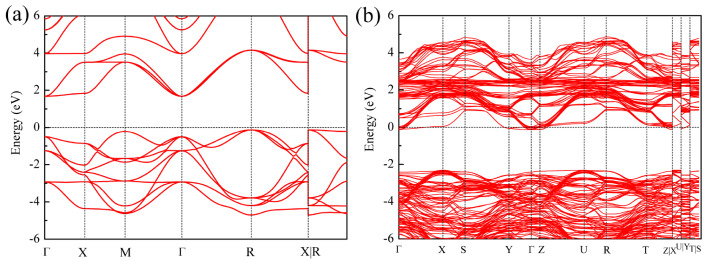
Band structure of ternary matrix SrTiO_3_ (**a**) and high-entropy CSBLPT (**b**).

The main origin of the performance differences in the mechanical and thermal properties of high-entropy CSBLPT and ternary matrix SrTiO_3_ depends on the different electronic interactions in their structures. To sufficiently understand the difference in properties between the two substances, the electronic bonding characteristics are investigated via the total density of states (TDOS), partial density of states (PDOS) and band structures, as shown in Figure 5 and Figure 6. It can be seen that the systems studied all exhibit semiconductor properties. For SrTiO_3_, the Fermi level is at the top of the valence band, the valence band maximum is at the R point and the conduction band minimum is at the Γ point, indicating an indirect band gap of 1.81 eV, which is consistent with the band gap of other calculation results [41]. Additionally, the PDOS indicates that the electronic contributions in the valence band for low energy levels are mainly from Sr-*d*, Ti-*d* and O-*p* orbitals. For the high-entropy CSBLPT in Figure 5b and Figure 6b, the DOS and band structure are more complex due to the random distribution of five different A-site cations; the conduction band moves to a lower energy level and the Fermi level is simultaneously shifted toward a higher energy level with the bottom of the conduction band, leading to band broadening and a slight increase in the indirect band gap to 2.10 eV. Furthermore, the Fermi level lies slightly above the conduction band minimum, indicating that high-entropy CSBLPT exhibits degenerate n-type semiconducting behavior with metal-like characteristics. This degeneracy arises from the substitution of divalent Sr^2+^ by trivalent La^3+^ at the A-site, which donates extra electrons and shifts the Fermi level into the conduction band. Also, the main electronic contributions near the Fermi level come from Ti-3*d* and La-5*d* orbitals. This result may facilitate carrier–phonon decoupling, beneficial for thermal barrier and thermoelectric applications [42].

Next, the bonding behavior in high-entropy CSBLPT and ternary matrix SrTiO_3_ is further described by quantitatively estimating the amount of charge transfer between atoms by Bader charges. The calculated average Bader charges for each atom are summarized in Table 6, obtained by subtracting the calculated charge from the number of valence electrons. It can be seen that the charge transfer occurs from the A-site atoms and Ti atoms to the O atoms, and the Ti atoms lose more electrons than the A-site atoms, indicating that the interaction in Ti–O bonds is stronger than that of A–O bonds. Moreover, the average charge values and Bader charge values of A-site atoms for high-entropy CSBLPT are greater than those of SrTiO_3_, implying that A–O bonds in CSBLPT are more ionic and the bond strength is higher than that of SrTiO_3_. In addition, the Bader charge values of cations co-occupying the A-site in high-entropy CSBLPT are different, suggesting different coordination environments for these cations, which further indicates that this may be responsible for the local lattice distortion and difference in thermo-mechanical properties.

## 4. Conclusions

In this paper, a high-entropy CSBLPT as a TBC material is investigated, and its structural, mechanical, thermal and electronic structure properties are investigated through the first-principles calculations in combination with special quasi-random structure. The obtained tolerance factor and atomic size differences reveal that the high-entropy CSBLPT enables the formation of cubic single-phase solid solutions, and the lattice parameters of CSBLPT perovskites match well with the relevant experimental data. The atomic random distribution and size difference in the A-site atoms lead to serious lattice distortion within the CSBLPT structure, and the results suggest that the influence of O atoms on lattice distortion is predominant, and the effect of the overall A-site atoms plays a distinctly greater role than that of the B-site atoms. The obtained elastic constants indicate that the substances studied are mechanically stable. The high-entropy CSBLPT is more suitable as a TBC material than ternary SrTiO_3_ due to a lower Young’s modulus, and the anisotropy index shows that the *E*_max_ of high-entropy CSBLPT appears along the [111] direction. By the obtained Debye temperature, the thermal expansion coefficient of the studied high-entropy CSBLPT is greater than that of SrTiO_3_. Moreover, the minimum thermal conductivity calculated with Clarke’s model and Cahill’s model shows that high-entropy CSBLPT has a lower thermal conductivity than SrTiO_3_, and the lowest minimum thermal conductivity occurs along the [100] direction. The electronic structure reveals that high-entropy CSBLPT is a degenerate n-type semiconductor with metallic characteristics, and the difference in the Bader charge also indicates the reason for the formation of structural lattice distortions. Therefore, high-entropy CSBLPT performs significantly better than SrTiO_3_ under working conditions and can be a promising candidate for TBC material.

## Figures and Tables

**Table 1:** The obtained equilibrium lattice parameters (Å), volume (Å^3^), tolerance factor t and atomic size differences δ (%) of high-entropy perovskite (Ca_0.2_Sr_0.2_Ba_0.2_La_0.2_Pb_0.2_)TiO_3_ and the corresponding ternary material.

	a	V	t	δ		a	V	t	δ
CSBLPT	3.913	59.91	1.004	6.73	BaTiO_3_	3.989	63.52	1.062	-
	3.913 ^a^	5.95 ^a^				3.979 ^b^			
SrTiO_3_	3.899	59.31	1.002	-	PbTiO_3_	3.925	60.47	1.019	-
	3.894 ^b^					3.93 ^e^			
	3.905 ^c^								
CaTiO_3_	3.849	57.02	0.966	-	LaTiO_3_	3.911	59.84	0.973	-
	3.851 ^d^					3.92 ^f^			

^a^ Ref. [16], ^c^ Ref. [27] from experimental results. ^b^ Ref. [28], ^d^ Ref. [29], ^e^ Ref. [30], ^f^ Ref. [31] from calculation results. *δ* is defined only for the multi-component A-site system (CSBLPT); it is not applicable to single-cation ternary perovskites (indicated by “-”).

**Table 2:** The ion sizes *r* and predicted average atomic displacements Δ*d* of each constituent element for high-entropy CSBLPT.

Type	Total A-Site	Ca	Sr	Ba	La	Pb	Ti	O
*r*	-	1.34	1.44	1.61	1.36	1.49	0.605	1.40
Δ*d*	0.056	0.125	0.016	0.020	0.032	0.086	0.031	0.081

**Table 3:** The elastic constants (GPa), elastic moduli (GPa), Poisson’s ratio *v*, Pugh’s ratio *G*/*B*, Zener anisotropic index *A*_Z_ and fracture toughness *K*_IC_ (MPa·m^1/2^) of high-entropy CSBLPT and ternary matrix SrTiO_3_.

	*C*_11_	*C*_12_	*C*_44_	*B*	*G*	*E*	*v*	*G*/*B*	*A_Z_*	*K*_IC_
CSBLPT	277.3	138.2	103.4	184.6	88.2	228.3	0.294	0.478	1.49	4.16
SrTiO_3_	348.8	103.2	113.7	185.1	117.3	290.5	0.238	0.634	0.93	2.91
	348 ^a^	101 ^a^	128 ^a^	184 ^a^						
	346.1 ^b^	101.3 ^b^	114.4 ^b^	183.0 ^b^	117.5 ^b^	290.4 ^b^	0.24 ^b^		0.93 ^b^	

^a^ Ref. [35] from experimental results. ^b^ Ref. [12] from calculation results.

**Table 4:** The calculated Shear and Young’s moduli (GPa) along the [100], [110] and [111] directions of high-entropy CSBLPT and ternary matrix SrTiO_3_.

	*G*			*E*		
	[100]	[110]	[111]	[100]	[110]	[111]
CSBLPT	103.4	69.6	80.8	185.3	237.1	261.5
SrTiO_3_	113.7	122.8	119.8	301.6	287.6	283.2

**Table 5:** The transverse *v*_t_, longitudinal *v*_l_, average sound velocities *v*_m_ (in m/s) and Debye temperature *Θ*_D_ (in K), as well as the theoretical minimum high-temperature thermal conductivity *κ*_min_ (in W·m^−1^·K^−1^) of high-entropy CSBLPT and ternary matrix SrTiO_3_.

Sample	*v*_t_	*v*_l_	*v*_m_	*Θ*_D_	$\kappa_{\min}^{Clarke}$	$\kappa_{\min}^{Cahill}$
CSBLPT	3819.5	7069.1	4263.1	554.7	1.409	1.563
SrTiO_3_	4847.5	8271.2	5374.4	694.9	1.728	1.923

**Table 6:** Average net charge of all elements in high-entropy CSBLPT and ternary matrix SrTiO_3_; unit of each atom is electron.

Sample	Sr	Ca	Ba	La	Pb	Ti	O
CSBLPT	1.56	1.59	1.52	2.06	1.37	2.06	−1.23
SrTiO_3_	1.61	-	-	-	-	2.06	−1.22

## Data Availability

The original contributions presented in this study are included in the article. Further inquiries can be directed to the corresponding author.
